# Trop-2 protein overexpression is an independent marker for predicting disease recurrence in endometrioid endometrial carcinoma

**DOI:** 10.1186/1472-6890-12-22

**Published:** 2012-11-14

**Authors:** Eliana Bignotti, Laura Zanotti, Stefano Calza, Marcella Falchetti, Silvia Lonardi, Antonella Ravaggi, Chiara Romani, Paola Todeschini, Elisabetta Bandiera, Renata A Tassi, Fabio Facchetti, Enrico Sartori, Sergio Pecorelli, Dana M Roque, Alessandro D Santin

**Affiliations:** 1 “Angelo Nocivelli” Institute of Molecular Medicine, Division of Gynecologic Oncology, University of Brescia, Viale Europa 11, 25123, Brescia, Italy; 2 Department of Biomedical Sciences and Biotechnology, Section of Medical Statistics and Biometry, University of Brescia, Viale Europa 11, 25123, Brescia, Italy; 3 Department of Medical Epidemiology and Biostatistics, Karolinska Institutet, SE-17177, Stockholm, Sweden; 4 Department of Pathology, University of Brescia, Viale Europa 11, 25123, Brescia, Italy; 5 Department of Obstetrics, Gynecology & Reproductive Sciences, Yale University School of Medicine, 333 Cedar Street, PO Box 208063, New Haven, CT 06520-8063, USA

**Keywords:** Endometrioid endometrial cancer, Trop-2, TACSTD2, Tissue microarrays, Prognostic factor

## Abstract

**Background:**

Endometrial cancer is the most common gynecologic malignancy in developed countries. Trop-2 is a glycoprotein involved in cellular signal transduction and is differentially overexpressed relative to normal tissue in a variety of human adenocarcinomas, including endometrioid endometrial carcinomas (EEC). Trop-2 overexpression has been proposed as a marker for biologically aggressive tumor phenotypes.

**Methods:**

Trop-2 protein expression was quantified using tissue microarrays consisting of formalin-fixed paraffin-embedded specimens from 118 patients who underwent surgical staging from 2001–9 by laparotomy for EEC. Clinicopathologic characteristics including age, stage, grade, lymphovascular space invasion, and medical comorbidities were correlated with immunostaining score. Univariate and multivariate analyses were performed for overall survival, disease-free survival, and progression-free survival in relation to clinical parameters and Trop-2 protein expression.

**Results:**

Clinical outcome data were available for 103 patients. Strong Trop-2 immunostaining was significantly associated with higher tumor grade (p=0.02) and cervical involvement (p<0.01). Univariate analyses showed a significant association with reduced disease-free survival (DFS) (p=0.01), and a trend towards significance for overall and progression-free survival (p=0.06 and p=0.05, respectively). Multivariate analyses revealed Trop-2 overexpression and advanced FIGO stage to be independent prognostic factors for poor DFS (p=0.04 and p <0.001, respectively).

**Conclusions:**

Trop-2 protein overexpression is significantly associated with higher tumor grade and serves as an independent prognostic factor for DFS in endometrioid endometrial cancer.

## Background

Endometrial cancer is the most common gynecologic malignancy in developed countries, with 47,130 new cases and 8,010 deaths projected for the United States alone in 2012 [1]. Endometrial carcinomas may be broadly dichotomized into two classes with distinct underlying molecular pathogenesis, clinical behavior, and histopathology [2]. Type I endometrial cancers comprise 80% of cases and are associated with a history of exposure to unopposed estrogen, favorable prognosis, and endometrioid histology (grade 1 or 2) [3,4]; mutations in k-Ras, PTEN, or mismatch repair mechanisms predominate [5,6]. Type II endometrial cancers constitute the minority of cases, but are typified by an aggressive clinical course with a distinct pattern of metastasis, older age at presentation, the absence of antecedent history of unopposed estrogen, and serous, clear cell or grade 3 endometrioid histology [7,8]; aneuploidy, mutations in p53, and Her2/Neu overexpression are common [9-12].

Trop-2 (also known as GA733-1, M1S1, EGP-1) is 35kDa transmembrane glycoprotein, encoded by the gene *TACSTD2* of chromosome 1p32. Trop-2 was originally identified in human trophoblasts [13] and has been shown to be upregulated in a variety of human carcinomas [14-20]. Importantly, overexpression relative to normal tissue has been documented in ovarian, endometrial, and cervical cancers across a broad range of histologies including papillary serous [21-23] and endometrioid [24] adenocarcinomas, squamous cell carcinomas [25] and carcinosarcomas [26].

In the era of personalized molecular medicine, the role of intrinsic tumor biology is increasingly recognized as a major determinant in clinical course [27]. There is growing evidence that Trop-2 overexpression portends adverse oncologic outcome [28-31]. This group has previously shown that strong expression as assessed by immunohistochemistry in epithelial ovarian cancers serves as an independent prognostic factor for decreased overall survival [32]. Recently, our group has also demonstrated that Trop-2 expression is significantly higher in endometrioid endometrial carcinomas (EEC) compared to normal tissues representing both proliferative and secretory phase endometrium, and that strength of immunohistochemistry staining correlates with increasing grade. Furthermore, compared to those that lack expression, EEC cell lines that overexpress Trop-2 are highly susceptible to antibody-dependent cellular cytotoxicity mediated by hRS7, a humanized monoclonal antibody against Trop-2 [24].

In this study, we perform secondary analyses of a sub-population from our previous work. We describe for the first time the clinicopathologic significance of Trop-2 overexpression in EEC using a large number of specimens via tissue microarrays (TMAs). This investigation holds important implications for patient care, understanding the contribution of Trop-2 to cancer pathogenesis, and further definition of the potential applications for therapy using hRS7 in the setting of treatment-refractory disease.

## Methods

### Endometrial carcinoma patients

The tumor tissue samples included in this investigation were derived from 118 patients sequentially treated at the Division of Gynecologic Oncology, University of Brescia, Italy between 2001 and 2009. The study has been performed following the Declaration of Helsinki set of principles and it has been approved by the Research Review Board- the Ethic Committee- of the Spedali Civili, Brescia, Italy (study reference number: 527/B4/4).

The inclusion criteria for the patients were the following: i) diagnosis of endometrial adenocarcinoma, endometrioid in histology, ii) informed consent obtained from the patient and iii) sufficient amount of tumor tissue in the FFPE block to create the TMA. All pathology specimens were reviewed in our institution and histological classification was performed according to WHO criteria, while pathological stage was determined in accordance with guidelines of the International Federation of Gynecologists and Obstetricians (FIGO). Tumor tissues were obtained from women undergoing total abdominal hysterectomy, bilateral salpingo-oophorectomy and peritoneal washings. Lymph node sampling or dissection was predominantly performed in patients with tumors characterized by deep myometrial invasion and/or high grade or aggressive histology. Obesity and advanced age were relative contraindications to full surgical staging. None of the patients had received preoperative chemotherapy or radiation. Age, stage, grade, medical comorbidities (e.g., diabetes, obesity, hypertension) and treatment information were recorded in all cases. A total of 103 patients had sufficient follow-up for inclusion in survival analyses.

### Tissue microarrays and immunohistochemistry

TMAs were created from 118 formalin-fixed, paraffin-embedded EEC tissues collected from the Department of Surgical Pathology, University of Brescia, Italy. TMAs were built using an automated tissue microarrayer (TMA Master, 3DHistech, Budapest, Hungary). Representative areas were chosen for sampling from haematoxylin and eosin (H&E) stained sections of selected normal endometrium (NE) and EEC cases. Four 0.6-mm cores were collected from different areas of each tumor block in order to overcome sample heterogeneity and the possible loss of tissue due to cutting. Separate TMAs were created for low and advanced tumor grade; normal tissue was included in all TMAs as an internal negative control. Four micron sections were cut from TMAs and H&E staining was used for confirmation of tumor tissue. TMA sections were subjected to antigen retrieval (40 min in water bath at 98°C in EDTA buffer pH 8.0) before application of the purified goat polyclonal antibody against the recombinant human Trop-2 extracellular domain (R&D Systems, Inc., Minneapolis, MN) diluted 1:100. The antibody was revealed with a biotinylated rabbit anti-goat (Vector Labs, Burlingame, CA), diluted 1:250, followed by HRP-streptavidin (Dako, Glostrup, Denmark) and diaminobenzidine (DAB) as chromogen; hematoxylin was used for counterstaining.

In the present investigation we used the immunohistochemical scoring method previously published by us in epithelial ovarian cancer [32]. Briefly, immunoreactivity was evaluated by three independent observers; membrane staining was graded for intensity (0-negative, 1-weak, 2-moderate, and 3-strong) and the percentage of positive cells was scored as 0 (0%), 1 (1-10%), 2 (11-50%), 3 (51-100%). A single immunohistochemistry (IHC) scale with scores 0–9 was obtained by multiplying the intensity and the percentage staining score and a total score was calculated grouping score 0 in total score 0, 1–3 in total score 1, 4 and 6 in total score 2 and 9 in total score 3. Digital images were resized by using Adobe Photoshop (Adobe Systems, Inc., San Jose, CA).

### Statistical analysis

The association between Trop-2 immunohistochemical staining, coded as 0, 1, 2 and 3, and clinical covariates were evaluated by non-parametric one-way ANOVA and Wilcoxon-Mann–Whitney tests. For survival analysis, three endpoints (cancer relapse, cancer progression, and death due to cancer) were used to calculate disease-free survival (DFS), progression-free survival (PFS) and overall survival (OS), respectively. DFS was defined as the time interval between the date of surgery and the date of identification of disease recurrence, PFS was defined as the time interval between the date of surgery and the date of identification of progressive disease (disease not treatable with curative intent) and OS was defined as the time interval between the date of surgery and the date of death. For all three endpoints the last date of follow-up was used for censored subjects. Survival models were fitted using the Cox proportional hazard models, while survival curves were drawn based on the Kaplan-Meier methods. The association of Trop-2 immunostaining with prognosis was evaluated. In all analyses, a p value < 0.05 was considered significant. All the analyses were performed using R (R Development Core Team, 2012) [33].

## Results

### Trop-2 immunostaining and clinicopathologic variables

The relationship between Trop-2 immunostaining and the clinical and pathologic features of the 118 endometrial carcinoma patients is shown in Table 1. Strong Trop-2 immunostaining was significantly associated with higher tumor grade (p=0.02), consistent with our previous results [24]. Moreover, Trop-2 protein overexpression was also significantly correlated with cervical involvement (p<0.01), and, marginally, with deeper myometrial invasion (p=0.08).

**Table 1:** Clinical and pathologic characteristics of 118 endometrioid endometrial cancer patients and their association to Trop-2 protein expression

	** Trop-2 protein expression**				
**Variable**	**n**	**Score≤1 n (%)**	**Score=2 n (%)**	**Score=3 n(%)**	p value*
*Age at diagnosis*					
<65	51	22 (43.1)	17 (33.3)	12 (23.5)	
					0.89
≥65	67	28 (41.8)	26 (38.8)	13 (19.4)	
*FIGO stage(2009 criteria)*					
I-II	86	37 (43)	33 (38.4)	16 (18.6)	
					0.56
III-IV	22	8 (36.4)	6 (27.3)	8 (36.4)	
Unknown	10	5 (50)	4 (40)	1 (10)	
*WHO grading*					
Grade 1	34	21 (61.8)	11 (32.4)	2 (5.9)	
					0.024
Grade 2-3	84	29 (34.5)	32 (38.1)	23 (27.4)	
*Myometrial invasion*					
M0+M1	55	26 (47.3)	22 (40)	7 (12.7)	
					0.08
M2	63	24 (38.1)	21 (33.3)	18 (28.6)	
*Lymph node status*					
Negative	79	35 (44.3)	28 (35.4)	16 (20.3)	
					0.69
Positive	15	5 (33.3)	5 (33.3)	5 (33.3)	
Unknown	24	10 (41.7)	10 (41.7)	4 (16.7)	
*Cervical involvement*					
Absent	83	37 (44.6)	33 (39.8)	13 (15.7)	
Glandular involvement	16	10 (62.5)	4 (25)	2 (12.5)	<0.01^$^
Stromal involvement	18	3 (16.7)	6 (33.3)	9 (50)	
Unknown	1	0 (0)	0 (0)	1 (100)	
*Adnexal involvement*					
Negative	108	45 (41.7)	42 (38.9)	21 (19.4)	0.97
Positive	9	5 (55.6)	0 (0)	4 (44.4)	
Unknown	1	0 (0)	1 (100)	0 (0)	
*Peritoneal cytology*					
Negative	100	40 (40)	37 (37)	23 (23)	
					0.44
Positive	8	4 (50)	2 (25)	2 (25)	
Unknown	10	6 (60)	4 (40)	0 (0)	
*Lymphovascular invasion*					
Absent	48	22 (45.8)	20 (41.7)	6 (12.5)	
					0.13
Present	56	21 (37.5)	20 (35.7)	15 (26.8)	
Unknown	14	7 (50)	3 (21.4)	4 (28.6)	
*Adjuvant Treatment*					
None	57	31 (54.4)	20 (35.1)	6 (10.5)	
Radiotherapy	36	12 (33.3)	13 (36.1)	11 (30.6)	
Chemotherapy	8	3 (37.5)	1 (12.5)	4 (50.0)	0.094^$^
Radiotherapy+Chemotherapy	9	2 (22.2)	5 (55.6)	2 (22.2)	
Unknown	8	5 (62.5)	2 (25.0)	1 (12.5)	
*Parity*					
Nulliparity	22	10 (45.5)	7 (31.8)	5 (22.7)	
					0.72
Multiparity	88	34 (38.6)	35 (39.8)	19 (21.6)	
Unknown	8	6 (75)	1 (12.5)	1 (12.5)	
*Body Mass Index*					
<25	36	12 (33.3)	14 (38.9)	10 (27.8)	
					0.58
≥25	64	27 (42.2)	24 (37.5)	13 (20.3)	
Unknown	18	11 (61.1)	5 (27.8)	2 (11.1)	
*Hypertension*					
Negative	48	18 (37.5)	18 (37.5)	12 (25)	
					0.26
Positive	63	27 (42.9)	24 (38.1)	12 (19)	
Unknown	7	5 (71.4)	1 (14.3)	1 (14.3)	
*Diabetes*					
Negative	96	38 (39.6)	39 (40.6)	19 (19.8)	
					0.95
Positive	15	8 (53.3)	2 (13.3)	5 (33.3)	
Unknown	7	4 (57.1)	2 (28.6)	1 (14.3)	
*Menopausal Status*					
Premenopausal	18	7 (38.9)	5 (27.8)	6 (33.3)	
					0.33
Postmenopausal	94	39 (41.5)	37 (39.4)	18 (19.1)	
Unknown	6	4 (66.7)	1 (16.7)	1 (16.7)	
*Smoking*					
Negative	82	29 (35.4)	35 (42.7)	18 (22)	
					0.91
Positive	20	9 (45)	6 (30)	5 (25)	
Unknown	16	12 (75)	2 (12.5)	2 (12.5)	

* Exact Wilcoxon-Mann–Whitney test.

^$^ Non-parametric ANOVA.

Unknown values were considered as missing and therefore excluded from the statistical analysis.

As previously reported by us, negative controls consisting in normal endometrial tissues demonstrated predominantly a weak immunoreactivity for Trop-2 (see Bignotti et al. 2011 [24] for figures representing Trop-2 immunostaining).

### Trop-2 immunostaining and patient survival

The median follow-up interval was 48.7 months (range 6.1 – 124.9 months). At the time of the last follow up, 86 patients (83.5%) were alive without evidence of disease, 1 patient (1%) was alive with disease, and 16 patients (15.5%) were dead of disease. The sites of recurrence were either local (pelvic lymph nodes, vaginal cupola), or distant (para-aortic lymph nodes, lung, brain, bone).

**Figure 1:**
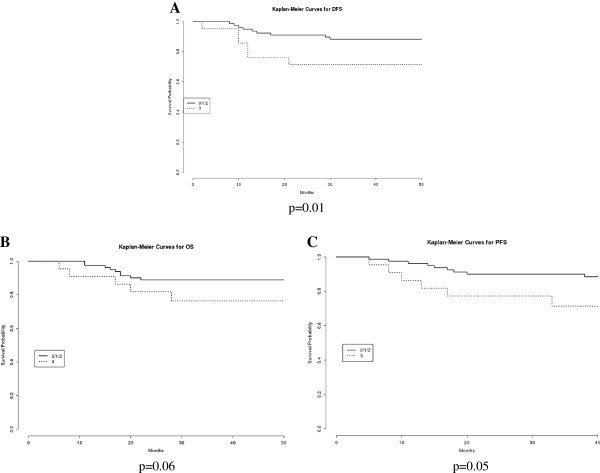
**Univariate survival analysis according to Trop-2 immunostaining on 103 EEC patients.** Kaplan-Meier survival curves displaying that stronger Trop-2 immunostaining (IHC score 3 versus IHC score 0/1/2) exhibits a significant association with reduced DFS (Figure 1A, p=0.01), while it shows a trend towards significance for overall and progression-free survival (Figure 1B and 1C, p=0.06 and p=0.05, respectively) on a cohort of 103 EEC patients.

As expected, known EEC clinical prognostic factors, such as FIGO stage and lymph node involvement, showed a statistically significant association with OS, PFS and DFS in univariate analysis (all p<0.01, Table 2). As displayed in Figure 1A and in Table 2, stronger Trop-2 immunostaining (IHC score 3 versus IHC score 0/1/2) showed a significant association with reduced DFS (p=0.01). Stronger Trop-2 immunostaining showed a trend towards significance for overall and progression-free survival (Figure 1B and 1C, p=0.06 and p=0.05, respectively). Trop-2 protein overexpression and advanced FIGO stage were identified as independent predictive factors for poor DFS (p=0.04 and p<0.001, respectively, Table 2) in multivariate analyses.

**Table 2:** Univariate and multivariate analyses of OS, DFS and PFS in relation to clinical parameters and Trop-2 protein expression

	**OS**				**DFS**				**PFS**			
**Variables**	**N=97**	**HR**	**95%CI**	**p**	**N=93**	**HR**	**95%CI**	**p**	**N=97**	**HR**	**95%CI**	**p**
***Univariate analysis***												
**Age**												
<65 vs ≥65	**103**	**1.23**	**0.46-3.28**	**0.68**	**93**	**1.06**	**0.41-2.75**	**0.90**	**102**	**1.37**	**0.49-3.79**	**0.54**
**FIGO stage**												
III-IV vs I-II	**97**	**17.05**	**5.47-53.17**	**<0.01**	**93**	**5.88**	**2.25-15.34**	**<0.01**	**96**	**16.73**	**5.28-53.03**	**<0.01**
**Tumor grade**												
G1 vs G2-G3	**103**	**0.001**	-*	**0.68**	**99**	**0.155**	**0.02-1.18**	**0.07**	**102**	**0.001**	-*	**0.69**
**Lymph node involvement**												
positive vs negative	**87**	**10.17**	**3.22-32.11**	**<0.01**	**85**	**6.05**	**2.09-17.51**	**<0.01**	**86**	**14.07**	**4.08-48.49**	**<0.01**
**Trop-2 IHC**												
score=3 vs score 0/1/2	**103**	**2.60**	**0.94-7.19**	**0.06**	**99**	**3.36**	**1.26-8.92**	**0.01**	**102**	**2.80**	**0.99-7.87**	**0.05**
***Multivariate analysis***												
**Trop-2 IHC**	**97**				**93**				**97**			
score=3 vs score 0/1/2		**1.83**	**0.65-5.18**	**0.25**		**2.82**	**1.05-7.58**	**0.04**		**1.76**	**0.63-4.88**	**0.28**
**FIGO stage**	**97**				**93**				**97**			
III-IV vs I-II		**15.94**	**5.08-50.04**	**<0.001**		**5.36**	**2.04-14.08**	**<0.001**		**16.73**	**5.31-52.73**	**<0.001**

*Because of the small number of events (deaths and progressions) in the G1 EEC category, the confidence interval was not reliable.

## Discussion

In this study, we used tissue microarrays to demonstrate that Trop-2 protein overexpression is significantly associated with higher tumor grade as well as cervical involvement and serves as an independent prognostic factor for DFS in EEC. A trend towards significance between Trop-2 expression and OS and PFS was exhibited and would likely have been achieved for OS with larger sample size or longer median follow-up, as greater time is required to demonstrate differences in OS compared to other endpoints [34]. Nevertheless, DFS has been shown to be highly correlated with OS and is increasingly received as an acceptable endpoint [35]. These findings are consistent with previous reports that suggest that Trop-2 overexpression identifies biologically aggressive phenotypes and poorly differentiated disease [24,28-31]. Though Trop-2 has been shown to be an excellent candidate antigen for targeted immunotherapy in multiple gynecologic malignancies including EEC [24], uterine papillary serous carcinomas [22], chemotherapy-resistant [23] and sensitive ovarian carcinomas [32], and cervical carcinomas [25], this is the first proof that Trop-2 overexpression can also prognosticate patient outcome in EEC.

The role of Trop-2 in cancer pathogenesis remains incompletely elucidated. In accordance with its original identification in trophoblasts, which possess the capacity to invade uterine decidua during the process of placental implantation, Trop-2 may analogously confer to cancer cells the capacity for proliferation and invasion [36,37]. Wang et al. (2008) [38] demonstrated the role of Trop-2 in anchorage-independent growth and the oncogenic potential of Trop-2 in colon cancer cells using both *in vitro* and *in vivo* models. Trop-2 has been shown to directly mediate tumor-associated calcium-mediated signal cascades [39] and activation of ERK 1/2-MAPK pathways [36], both of which govern cell cycle progression [40,41] and may protect cancer cells from apoptosis [42]. A highly conserved phosphatidylinositol 4,5-bisphosphonate (PIP_2_) binding sequence that overlaps with a protein kinase C phosphorylation sites within the cytoplasmic tail suggests a specific role in signal transduction [43,44]. Recently, Trerotola et al. [45] demonstrated that Trop-2 upregulation is necessary and sufficient to promote cell growth in different cancer cell types and that its somatic knockdown is able to extinguish tumor cell growth. The Trop-2 transcription control network, including factors activating Trop-2 expression or downstream growth-stimulatory pathways has been recently reported [46]. Interestingly, Trop-2-cyclin D1 chimeric mRNAs have also been identified in a variety of tumors and appear to lend enhanced stability to cyclin D1, resulting in cellular immortalization [47] and possibly prevention of anoikis [48]. Of note, Trop-2 expression appears to discriminate populations with stem-like activity [49], which may be important for initiation and perpetuation of cancers [50,51]. A different role of Trop-2 in cancer growth has been recently reported by Lin et al. [52] in lung carcinoma, where Trop-2 is epigenetically downregulated and affected by LOH, resulting in cancer progression via activation of IGF-1R signalling.

In this report, we provide immunostaining results indicative of protein expression. Immunohistochemistry is widely accepted as an adjunct technique to routine histologic analysis to offer additional diagnostic and prognostic information [53]. We did not evaluate Trop-2 at the mRNA level since transcript copy number and protein expression are poorly concordant for this antigen [32]. This phenomenon is not uncommon; genomic analyses at best are believed to capture only 40% of proteomic variations due to post-transcriptional regulatory mechanisms in mammalian cells [54].

Generally accepted prognostic factors for endometrial carcinoma are nodal involvement/stage, grade, and histologic subtype [55]. As anticipated, in this cohort Trop-2 overexpression correlated with grade and propensity for cervical involvement. Tumor location within the uterus has potential clinical relevance, since the lymphatic drainage of the cervix differs from that of the fundus in that it is much more rich and tends to involve the pelvic as opposed to the para-aortic nodes [56].

Many recent attempts to discover novel prognostic factors for endometrial cancer have been discouraging. Koyuncuoglu et al. (2012) [57] described the loss of e-cadherin expression in association with advanced stage and poor differentiation in both EEC and non-EEC, but failed to demonstrate statistical significance between e-cadherin levels and survival using multivariate analyses. Similarly, while Mhawech-Fauceglia et al. (2012) [58] noted a high percentage of uterine papillary serous and EEC cases with expression of the tight junction protein claudin-7 and the cytoskeletal protein moesin, no association existed between these proteins and overall or disease-free survival.

## Conclusions

In summary, we provide the first demonstration that Trop-2 overexpression prognosticates poor disease-free survival in EEC. This study corroborates previous reports on the importance of Trop-2 expression in human tumors to stratify biologically aggressive disease variants. Importantly, because Trop-2 overexpression may represent an independent prognostic factor for earlier cancer recurrence, its determination in EEC tissue samples may be clinically useful in the attempt to identify patients at higher risk of relapse before surgery and, subsequently, to optimize follow up and adjuvant treatments. These data, however, should be confirmed in additional studies on larger patient cohorts before routine Trop-2 IHC evaluation may be applied in the clinical setting.

## Competing interests

The authors declare that they have no competing interests.

## Authors’ contributions

ADS conceived, coordinated, designed the study, interpreted the data and revised the manuscript. EB participated in the study design, created the patients’database, reviewed medical records, interpreted the data, drafted and wrote the report. LZ helped in reviewing the medical records and in drafting the manuscript. SC performed statistical analyses, created the tables, interpreted the data and helped to draft the manuscript. SL and MF performed pathological and immunohistochemical study. AR supervised the research group and critically reviewed the manuscript. EB and CR helped in collecting data from medical records and critically reviewed the manuscript. PT and RAT helped in collecting follow-up data and critically review the manuscript. FF coordinated the immunohistochemical study. ES partecipated in the study design, interpreted the data and critically reviewed the manuscript. SP provided funds and participated in the design of the study. DR helped in drafting the manuscript discussion and critically review the paper. All of the authors read and approved the final manuscript.

## Pre-publication history

The pre-publication history for this paper can be accessed here:

http://www.biomedcentral.com/1472-6890/12/22/prepub
